# The evaluation of research papers in the XXI century. The *Open Peer Discussion* system of the *World Economics Association*

**DOI:** 10.3389/fncom.2012.00054

**Published:** 2012-08-07

**Authors:** Grazia Ietto-Gillies

**Affiliations:** ^1^Centre for International Business Studies, London South Bank UniversityLondon, UK; ^2^International Business, Department of Management, Birkbeck University of LondonLondon, UK

**Keywords:** research evaluation, academic journals, open peer review, economics, World Economics Association

## Abstract

The paper starts with a brief discussion of the traditional peer review (TPR) system of research evaluation, its role, and the criticisms levelled at it. An analysis of specific problems in economics leads to a full discussion of the Open Peer Review (OPR) system developed by the World Economics Association (WEA) and the principles behind it. The system is open in the following two respects: (a) disclosure of names of authors and reviewers; and (b) inclusivity of potential reviewers in terms of paradigmatic approaches, country, and community. The paper then discusses the applicability of the same system to other disciplines. In doing so, it stressed the aims of various evaluation systems and the possible pitfalls of rating systems. It also speculates on the future of journal publication.

## Introduction

The peer review (PR) system of research evaluation used for many decades in journal publications has increasingly come under criticism. In the traditional peer review (TPR)^1^ system a small number of reviewers appointed by the editor write reports that form the basis for the decision to publish or not. The names of reviewers are usually kept secret from the authors; the practice on the anonymity of authors varies from journal to journal and indeed between disciplines.

Most criticisms of TPR concentrate on the following^2^: (a) efficiency issues and in particular the high and increasing social costs for the academic community and the length of the publication process (Campanario, 1998a,b; Ginsparg, 2002; Frey and Osterloh, 2007); (b) pressure on authors to accept the suggestions of reviewers—even when they do not agree with them—in order to have their paper published (Frey, 2003); (c) low effectiveness in terms of quality assurance such as the detection of errors or of plagiarism or the weeding out of very poor research (Campanario, 1998a; Bedeian, 2004); and (d) difficulty in identifying ground-breaking research (Horrobin, 1990; Gans and Shepherd, 1994; Campanario, 1995; Gillies, 2008).

There are, nonetheless, many supporters of the TPR system among academics (Ledeberg, 1978; Garfield, 1986; Legendre, 1995). They claim that, though the system does have some faults, it is the best available and one on which there is the widest consensus about its fairness. This view is largely shared by the Report on the inquiry of the House of Commons Science and Technology Committee (2011).

Most of the problems of the TPR have been known for a long time. It is legitimate to ask ourselves why they have come to the fore now. I suggest that this is due to the following reasons. First, the fact that there has been an increase in assessment in general and researchers are beginning to ask whether it is all necessary and indeed whether this type of culture encourages academic endeavors. Second, there has been an increase in the number of journals and in the number of papers seeking publication—and thus journal space—in response to the widening assessment culture. This proliferation of papers and journals is leading to increasing reviewing work and, indeed, to overload for many reviewers. A third—and in my view most relevant—factor is that the power of digital technologies is making the old system redundant. Essentially, what I am saying is that—whether the commentators realize it or not—our critical attitude to TPR is emerging because *there is a way out*. It is on the basis of this last point—the existence of a way out—that the new system of evaluation—the *Open Peer Discussion* system—in economics was developed as discussed in section “The WEA Evaluation System: Basic Principles and Process.”

However, whether a new system can replace the TPR one largely depends on what we expect from an efficient and effective evaluation system. Most researchers expect it to perform the following functions. (i) Quality assurance for the readers and guidance as regard fields of specialization. (ii) Help in improving the research paper. (iii) Guidance to editors in the allocation of limited journal space.

Regarding (iii), unfortunately the TPR system is known to have led to some perverse allocation: the rejection of papers containing fundamental research. Several instances from the history of science modern and past and in several disciplines have come to light (Horrobin, 1990; Gans and Shepherd, 1994; Campanario, 1995). Closer to us, The Guardian (2011) reports that the groundbreaking research of Daniel Shechtman—the 2011 winner of the Nobel Prize for Chemistry—was, at first, rejected by peers and he was asked to leave his research group to which he was, allegedly, bringing disgrace by his theory and findings. Gillies (2008) gives a philosophical reason—based on an application of Kuhn to the research assessment field—of why it should be so. He claims that the TPR system is likely to favor orthodox research, the type of research that operates competently within a well-established and majority paradigm rather than research which is ground-breaking. Yet, the history of science shows that, while the former type of research may be relevant, it is the ground-breaking research that gives science, the economy, and society the best returns in the long run. Sir James Black, the 1988 Nobel Prize winner for medicine, did not mince his words regarding the impact of TPR system on innovative research. In a Financial Times (2009) interview he is attributed the following statement: “The anonymous peer review process is the enemy of scientific creativity …. Peer reviewers go for orthodoxy ….”

The next section considers the specific problems of research evaluation in economics and the establishment of the *World Economics Association* (WEA). Section “The WEA Evaluation System: Basic Principles and Process” presents a PR system developed by the WEA and designed to overcome some of these problems. The last section discusses the applicability of the WEA system to other disciplines and emphasizes the desirability to consider the aim of evaluation in developing alternative systems.

## Specific problems in economics. The WEA

Economics, the dismal science, is also among the most problematic of sciences in terms of research evaluation. The TPR system has been applied in economics as well as—and as long as—in most other sciences—natural or social—and in the humanities. It has drawn a similar amount of criticism.

However, in economics there are also problems that are largely specific to the subject and are additional to the general problems of TPR. Here are some of these specific problems.

First, in economics there is, usually, co-existence of several schools/paradigms contemporaneously. This is one of the features that differentiate the social from the natural sciences according to the philosopher of science Thomas Kuhn^3^. Second, economics and its theories tend to be closely linked to political ideologies and it is this aspect that makes it possible and desirable to have co-existence of several paradigms. Ideology plays a role in the type of issues considered by researchers and economists in general; in how they characterize the operations of the economic system; which methods and empirics—if any—they use to corroborate their theories; and in how they interpret their results^4^. The third problem—common to other sciences—is that it is possible to earn large amounts of money outside academe as advisers to politicians and consultant for large businesses and institutions. The contact with the real world of business and policy-making may help in the understanding of economic issues and in the development of research; however, it may also affect the objectivity of the researcher. In terms of evaluation of research papers, the referees themselves may be—even unconsciously—biased in favor or against research that is too closely linked to the business or politics they are involved in.

Regarding the first issue—the co-existence of several paradigms—the following alternative paradigms/schools can be identified in economics: Keynesian, Marxist, Sraffian/neo Ricardian, Austrian, institutionalist, and neoclassical. The latter school is the one most closely associated with the following features: supremacy of the market and of its price mechanism as allocator of resources; equilibrium analysis; disregard for uncertainty in economic processes. After the Second World War the Keynesian, neoclassical and Marxist schools were the main paradigms across the western world. To a large extent they coexisted though the Marxist school was always a minority one. It is, however, interesting to note that in those early decades after WWII most economists, whatever the school they belonged to, seemed to accept Keynesian analysis and its policy prescriptions: government intervention to smooth the trade cycle was widely accepted. The Keynesian theory was, in fact, adapted by the neoclassical school to fit in with their equilibrium analysis in the so-called neoclassical synthesis.

In the last 30 years two major changes have occurred in economics. There has been a move toward less pluralism and toward the dominance of the neoclassical school. Moreover, the now prevailing neoclassical school has changed its character compared to its earlier, traditional form. An extreme form of neoclassical economics has now become the dominant paradigm in economics; one with the following features. It: (a) rejects Keynesian analysis and policies; (b) gives the market a supremacy role linked to the belief that unfettered markets can deliver equilibrium and stability; and hence (c) rejects the role of governments in regulating markets^5^. This extreme form of neoclassical economics corresponds, in politics, to the ideology of neoliberalism. As the latter ideology prevailed, so did the supremacy of the neoclassical paradigm in all aspects of economic life and of economics as a discipline; from journal publications to university and school curricula to media analysis and to policy recommendations. Gradually all other paradigms have been marginalized—though not obliterated—and the neo-classical one has become the mainstream paradigm and almost the only one prevailing in terms of policy recommendations.

The TPR system of research evaluation has been one of the key elements in helping the neoclassical system achieve supremacy and making economics almost a single paradigmatic subject. There is an interaction at work: within the TPR system of research evaluation a discipline with predominance of a single paradigm will tend to favor publication of papers—particularly in the highest rated journals—within that paradigm^6^. This is largely because most reviewers belong to the mainstream paradigm and are very likely to see negatively papers developed in the context of alternative paradigms. This outcome may not necessarily be the result of deliberate strategies to cut out other paradigms neither of poor judgment: it may, in many cases, be the result of being confronted with something unfamiliar and which, therefore, appears to be not quite right. It must be remembered that many economists currently younger than 50 or so years, may not have been taught any of the alternative paradigms particularly if they have been to very prestigious universities. Moreover, once a paradigm starts prevailing and monopolizing the top journals as well as the allocation of research funds and jobs, more and more young researchers will work within it thus consolidating its supremacy.

Dissatisfaction with this situation and with the dominant economics paradigm—and with the policies it led to—was bound to develop^7^. It has, indeed, increased following the financial crisis of 2008 when the economics profession has come under justified attacks for: (a) having encouraged disastrous economic policies, particularly with regard to financial deregulation; and (b) being at a loss as to what to do once the crisis manifested. Since then the economic situation has worsened and criticisms of the subject and of the profession as a whole continue. It should, however, be noted that the policies of most governments were inspired by main stream type of economics. There were quite a few economists who had been warning against excessive financial deregulation and marketization of economies as they are warning now about promoting deflationary policies in the context of a recession. According to the Keynesian paradigm deflationary policies in the context of low effective demand (for consumption, investment, exports, and government expenditure) lead to lower state revenue and thus they exacerbate the problem of governments' debts. But, alas! these Cassandra voices are not heeded and the relevant papers rarely find their way into prestigious journals or policy circles.

The paradigmatic dominance^8^ in the main journals led to concomitant dissatisfaction with the TPR system of research evaluation. The problems were further complicated by the fact that the mainstream paradigm was seen as associated with the dominance of Anglo American economics and economic policies. The *American Economic Association* (circa 17,000 members) and the old and prestigious *Royal Economic Society* (c. 3300 members) were seen to dominate the type of economics being taught in universities all over the world, the most prestigious journals and—indirectly—the top jobs in finance, politics, and business economics. It also dominated and still dominates the policies of many governments in both developed and developing countries.

It is in this context that the WEA was established. The brainchild of Edward Fullbrook, the WEA was developed with the collaborative effort of a few other people^9^ from different parts of the world. All work is done on a voluntary basis by committed people. It was launched on 16th May 2011^10^ and within a year it reached a membership of approx 10,000. Membership is free and donations are encouraged.

The WEA aims (www.worldeconomicsassociation.org) include: plurality of approaches to economics; inclusivity of economists from every part of the world and from every persuasion; commitment to high-level research and to the full utilization of the digital technologies. Its main activities—all online and free to members—are the management of three journals and of conferences. More journals may be developed in the future.

## The WEA evaluation system: basic principles and process

The initiators of the WEA are fully committed to high-level research and to research evaluation. However, they consider that the aims of plurality of approaches to economics and inclusivity could not be achieved within the operation of the TPR system of research evaluation for the reasons explained in section “Specific Problems in Economics. The WEA”. They therefore developed a different system of research evaluation^11^ to be used by two of its journals in alternative to the PR system. The journals are: *World Economic Review (WER)* and *Economic Thought: History, Philosophy and Methodology (ET)*. The third journal of the Association the *Real World Economics Review (RWER)* has been in operation for several years and is now incorporated into the WEA umbrella. It publishes articles on economic, political, and social issues of wider appeal—and for a wider readership—than the more specialized economics field of the other two journals. The papers are evaluated by the editor of the RWER who publishes what he considers appropriate and after an editing process. The system used in the WER and ET is based on the following principles.

- PR is a very useful system for research evaluation and development. However, the digital technologies have made journal space allocation an irrelevancy. It is therefore possible to decouple the dissemination/publication function^12^ from the evaluation and development function of PR.
- The digital technologies are being used extensively by journals' publishers in the publication process. For example in communications between editors, referees, and authors and in copy editing. However, so far, little use has been made of them for the *evaluation process itself*.
- The TPR system is based on the principles of assessment/rating and of exclusion. Because journal space is limited and the ratio of paper submission to acceptances is very high, the editors necessarily look for support and justification for the rejection of many submitted papers. In order to do so, reviewers often look for faults rather than areas which are positive and could be further developed. These critical points do not mean to devalue the work of reviewers^13^—many of whom labor very hard and often come up with helpful suggestions—but only to point out a problem in the system they are caught in: in the end no matter how helpful some of them may want to be, their reports are used to exclude papers from publication in specific journals. But again no blame can be attached to the editors who have to allocate limited space in their journals.
- Research can achieve best results when it is developed as a social activity^14^ not necessarily in the sense of two or three people working together on a project, though this is, increasingly, the case in many fields. The social context is seen here as researchers developing their own ideas on the basis of previous research—which is always the case—and benefiting from discussions and interchanges with peers in a constructive environment. The involvement of peers in the evaluation and further development of research is very useful. However, it does not have to be on a confrontational and rating basis. It can take place on the basis of exchange of ideas for the advancement of the specific topic of the paper.
- The involvement of many researchers in the evaluation process is preferable to only 2–3 reviewers because: (a) the large number of reviewers—from an inclusive constituency—is more likely to contain a few who can spot plagiarism, mistakes, data problems; (b) if many people—belonging to several paradigmatic approaches and several countries and communities—read a paper it is more likely that one or two of them spot the originality and value of a paper which is out of the ordinary and may thus appear strange and wrong to most researchers. Thus, one of the major pitfalls of the PR system is less likely to manifest. Moreover, the involvement of many commentators increases the likelihood of researchers belonging to different schools/paradigms contributing. One of the major problems in economics research and publication can, therefore, be avoided.
- Double-sided openness: the names of the author(s) and those of reviewers are revealed. The attribution of comments to a specific paper encourages commentators to come forward with their views knowing that they are posted with their names. Attribution may, therefore, eliminate reticence in putting forward very original comments. Attribution may also encourage commentators to consider carefully their critical arguments and make sure that they are not inspired mainly by adherence to a specific paradigm and ideology.
- A common worry about open posting (where the names of authors and commentators are disclosed) is that commentators feel embarrassed to be critical. However, it is worth pointing out that: (a) reviewers of books—where a doubly open system is used as a matter of normal academic activity—are often quite critical; (b) moreover, if the process is online, commentators, and authors may be in very distant parts of the world and do not know each other; and (c) if the system is less confrontational than the TPR system this is no bad thing: a critically positive system is more likely to lead to the advancement of research.
- Post-publication evaluation is as important for the advancement of research as pre-publication one. The life of a paper does not end with publication; hopefully that is only its beginning. Other researchers will read the paper for years to come; the continuing readership success of the paper through time is evidence of its relevance. Some readers may develop further research of their own after reading an article and their research may lead to new publications in their own name. However, others may have points to make about it which do not amount to the development of a full research project or paper but that can, nonetheless, be relevant and useful for the further advancement of the field. A *post-publication commentary* as a standard feature of journals allows these people to have their comments published—at the discretion of the editors—with attribution.

The above principles inform the WEA system of *Open Peer Discussion (OPD)* whose actual process is the following^15^.

1. Papers submitted to the journal are first vetted by the editors. Those that meet minimum standards of professional quality are posted with the name of the author on the journal's *Discussion Forum (DF)*. Each (DF) remains open for eight weeks from the posting of the paper. All members of the WEA have access to the DF and can actively participate in it.
2. Comments on the posted paper are invited from the membership as well as solicited by the editors from experts in the field. Names of possible commentators may also be suggested by the authors. The comments are screened by the editors and then posted with the name of the commentator unless anonymity is requested. The authors can respond to the comments and their response will be posted with attribution.
3. Once the DF is closed the editors reach their decision on whether to publish the paper and—if accepted—the author is invited to review the paper for publication. Selected important reviews will be published at the end of the paper with prior agreement from the commentators.
4. A *Post-Publication Commentary* section is open on the journal. Post-publication comments are sent to the editors who will decide whether to post them or not.

## From the specific to the general. A vision for future evaluation systems

The previous two sections presented an application of an Open Peer Review (OPR) system to the case of economics. To what extent can this specific case be generalized to apply to other disciplines? In order to answer this question let us consider what elements are necessary for the system to operate and whether those elements can be had in other disciplines.

The system is open in two respects: (1) because there is attribution of authorship for both authors and reviewers; and (2) because there is inclusivity of potential reviewers in terms of paradigmatic approaches, countries, and communities. Point (2) requires (a) the use of digital technologies; and (b) the full involvement and empowerment of the research community in any specific field. In order to realize point (b) it is necessary to be inclusive and thus to reach a large number of diverse potential reviewers. This is now possible via the digital technologies which, therefore, enter into the very process of research evaluation rather than contribute only via the digitalization of administrative functions. Point (1) is more likely to lead to reviewing that is: more carefully thought through; less likely to be biased and more likely to lead to comments that make positive points towards the development of research. Point (2b) raises the probability of the reviewers being able to spot errors, fraud or ground breaking research.

**Figure 1:**
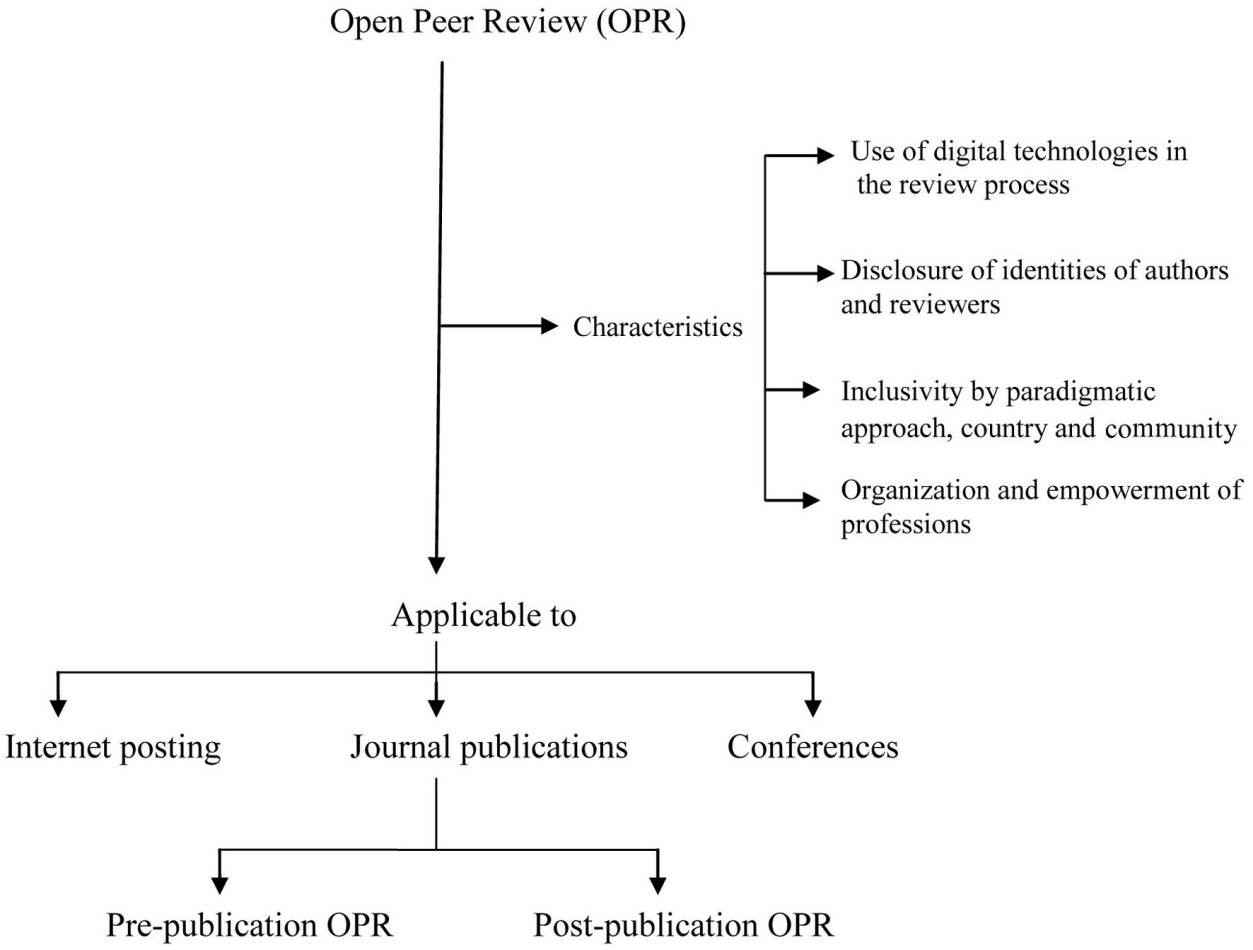
**Open Peer Review.** Characteristics and applicability. Developed from Figure 2 in Ietto-Gillies (2012).

Figure 1 illustrates the elements on which the OPR system is based as well as its possible applications: to journal publications, to internet posting and to online conferences. These requirements can be had for all or most disciplines and therefore I see the possibility of applying the OPR system discussed above to fields other than economics. If disciplines are very large in terms of members—as is, indeed, economics—it may be necessary to classify the members by fields of specific interest. Participation to the OPR process would then be limited to researchers that specialize in the field of the paper to be reviewed.

It should, however, be noted that—quite independently of the discipline—just the reaching of many diverse researchers is no guarantee of having a large and diverse contribution to OPR. In fact early experiences^16^—including that of the WEA—point to the fact that researchers are timid about exposing themselves as authors or reviewers. This is not surprising given the culture of secrecy in PR to which we are all used. It will take time, but I believe that a change in culture is possible. Meanwhile editors may want to consider starting the system with a mixture of anonymous and attributed reviews and may, generally, be prepared to be flexible in terms of allowing anonymity in special cases.

How do we progress from current positions toward the implementation of OPR systems? I see various possible routes. First, a bottom up approach; this is the one used in the WEA case and the only route of which I have direct experience. Volunteers within the discipline work toward the establishment of a new association with the specific objectives of organizing online journals and conferences. This initial process involves a considerable amount of work, commitment, and goodwill. Alongside efforts to increase the membership and promote the activities, there will be efforts to set up the activities such as: appointing editorial boards and editors and producing tight guidelines for both conferences and journals. A second route would be to start from existing associations and propose to members OPR-led activities. A third route is to start from existing journals and encourage the readership to opt for OPR processes and also to participate in these processes as authors and reviewers.

Though, in this writer's view, the system is not discipline-specific it does have boundaries in respect to other elements. First, in terms of aims. The main aim of the system presented in this paper is to contribute to the development of research. However, the reviewing process may be developed—with the aid of digital technologies—to meet other aims. For example, to help readers—who may or may not themselves be researchers—to find their way through large number of published works. There are several initiatives in this direction such as the Faculty of 1000^17^ for the biological sciences. Similar aims are behind the development of quick, snappy ratings of papers, a practice that is spreading fast. Personally I am not in favor of these types of rating evaluations: they stress the competitive side of research rather than the collaborative and social nature of research and, moreover, they lend themselves to abuse and to possible misinterpretations by the readers. The digital technologies offer us many possibilities for rating purposes and we are in danger of developing more and more rating systems just because the technology allows us to. In other words we are in danger of being technology-led rather than aim-led with the technology being used to meet specific aims. Whether we are in favor of rating or not, in my view the key question to ask ourselves is: what aim do we want to achieve by rating? How can the technology help us to achieve those aims?

The possible developments in PR systems discussed in this paper and, indeed, in this journal issue speak for a future evaluation system different from the current one. Moreover, if we consider the combination of open access (OA) systems in the field of dissemination and of OPR system in the evaluation field^18^ we may be led to speculations about the future of publication via journals. In the discourse on evaluation—including the OPR system presented above—the starting point is publication and how to develop an alternative system of evaluating papers pre- and post-publication.

However, let us put “evaluation with the aim of development” center stage and let us assume that some system of OPR becomes widespread. We can then speculate whether in such a future we shall still need journals—be they in electronic or paper version. We shall still need “editors” to manage the evaluation function. However, once the paper has been openly reviewed and revised and receives the approval of the editors, do we need it to be bundled up with other papers and be published as part of a journals issue? What are the benefits of such bundling and publication process? Could it not just be posted on an OA repository labeled something like “evaluated and revised papers”? It might still be possible to have comments on these finalized papers and even have the authors write “Addendums” to their papers if they later want to make further developments to it. Regarding the bundling together in a journal issue, might there still be scope for this practice but in terms of bundling up by topic? Might readers find it more useful to have papers bundled up by topic rather than by the date at which various papers happen to be ready for publication?

I do not have answers to many of these questions. The field and the issues are evolving. The only thing I am sure of is that the future of dissemination and evaluation of research will look very different from the present.

### Conflict of interest statement

The author declares that the research was conducted in the absence of any commercial or financial relationships that could be construed as a potential conflict of interest.

## Footnotes

^1^Peer review (PR) means review by experts and this includes a variety of systems. For this reason I prefer to distinguish between PR and TPR.

^2^These issues are discussed at greater length in Ietto-Gillies (2010). See also Kravitz and Baker (2011) and Birukou et al. (2011) in this issue.

^3^See Gillies (2012) for an account of Kuhn's position on the social versus natural sciences.

^4^The link between economics and ideology does not mean that it is impossible to reach conclusions regarding the validity of results and the corroboration of theories. Usually, the corroborating or refuting evidence builds up and ideologies can be set aside.

^5^This does not prevent big business and the financial sector asking for government support when they are in trouble.

^6^Gillies (2012) gives a philosophical and mathematical justification of why and how the TPR system of research evaluation in a discipline with prevalence of one paradigm will lead to the highest rating for research in that specific paradigm. Lee (2007) give a statistical analysis of the relationship between scores of journals and adherence to mainstream or minority paradigms. It shows that the highest-rated journals shy away from publishing research papers developed within alternative paradigms.

^7^It has led to the Post-Autistic Economics movement (Fullbrook, 2003) and to the Association of Heterodox Economists (Lee, 2008).

^8^The problems of paradigmatic dominance and power structure are considered in Bachmann (2011) in this issue.

^9^Including the author of this article who contributed, in particular, to the evaluation system for the WEA journals and to the development of its system of online conferences.

^10^Its legal status is of a Community Interest Company.

^11^Most of the points in the WEA alternative system of research evaluation have its origin in Ietto-Gillies (2008 and 2010).

^12^See Priem and Hemminger (2012) in this issue.

^13^This author has been associate editor of an academic journal—*Transnational Corporations*—and has been involved for many years in TPR activity as a reviewer and as an author. In the latter activity she has received many helpful comments and some lousy ones.

^14^Lee (2012)'s approach in this issue is also based on the principle that open, attributed reviews by many researchers can help to further develop a specific paper. However, the recommended system is different from the one presented here.

^15^Some of the following elements of OPR are applied also to the WEA conferences in which the text-based discussion takes place online over a four weeks period.

^16^See Pöschl (2012) in this issue. The Science and Technology Committee (2011) report the successful case of the *British Medical Journal* and the unsuccessful one of *Nature* (p. 26, para 23).

^17^www.f1000.com

^18^The relationship between OA and OPR is discussed further in Ietto-Gillies (2012).
